# Remarks on Scurvy as Observed in Cumberland, and the Southern Parts of Scotland

**Published:** 1847-10

**Authors:** Henry Lonsdale

**Affiliations:** Physician to the Cumberland Infirmary, &c., &c.


*Remarks on Scurvy as observed in Cumberland, and the Southern parts of Scotland.* By Henry Lonsdale, M. D., F. R. C. P. Ed., Physician to the Cumberland Infirmary, &c., &c.

The history of scurvy, interesting to the physician and no less gratifying to the philanthropist, is often cited to show the advantage of well-directed hygiene, and the superiority of modern medicines over that of less favoured times. Though very few practitioners of the present day have seen scurvy, all seem pretty nearly agreed that the disease has its origin in the absence of fresh vegetables, and that a restoration to these, or the free use of fruits of the genus *citrus* of the *aurantiaceæ*, will speedily establish health in scorbutic patients. As medicine is a progressive art, and men are now taught the advantage of viewing things in a broader light, it need not surprise us to see dogmas set aside, and principles, apparently well established, called in question by a wider and more searching philosophy. Much praise is due those who set themselves the noble task of observing disease *de novo*; even when, as in scurvy, history to all appearance had completed its task. I am led to these remarks from a perusal of the opinions published in the last number of this Journal by my esteemed friend and preceptor, Professor Christison, who, not content with the views of approved authors on the subject of scurvy, has attempted a further generalization, or at least introduced some novel and curious facts bearing significantly on any discussion which may hereafter be entered upon in relation to this disease.

During the past winter, and more especially about the beginning of the present year, I frequently observed amongst the out-patients of the Cumberland Infirmary, and occasionally in those of better circumstances in respect to food and clothing, a deteriorated condition of the system with no very specific, or localizable disease. The parties presented an unhealthy complexion, and complained of great languor, listlessness, and debility, irregular dyspeptic symptoms, diarrhœa, and more frequently dysentery. The legs were sometimes swollen, œdematous and painful, and the most trifling sores were slow to heal. Rheumatic pains were common, and in women of less robust habit menorrhagia was not unfrequent. These and other ailments became associated in my mind with what the older authors would have termed a "cachectic condition."*

Depletives of all kinds could not be borne; tonic medicines on the other hand were in great request, and fulfilled the best indications. On conversing with Mr. Thomas, our active house surgeon, on the prevalence of disease and its peculiarities, during the month of March, he expressed his doubts as to the character of a few cases where the parties complained of contusions, sprained ankles, &c., and gave me reason to apprehend that scurvy had shown itself amongst the applicants for *surgical* relief. At that time I had not seen scurvy, nor did any come under my notice till the 20th of April, when a girl of 18 years of age applied as an out-patient. Three days subsequently, two railway labourers were brought to the infirmary from the "Summit Huts" on the Caledonian Railway, a distance of nearly fifty miles. One of these men, aged forty-three years, rapidly fell a victim to the disease. I abbreviate the house surgeon's report of the case.

His face is swollen—lips livid—ecchymosis about the right eye. Such was his difficulty of breathing, that when carried from the conveyance into the Infirmary, he could not walk above two or three steps. The sounds of the heart were feeble, and a short bruit accompanied the diastole: respiratory sounds, healthy on the anterior and lateral parts of chest, but inaudible behind. This man, who gave but an imperfect account of himself, said he had been able to work until about a fortnight ago, when his illness commenced with difficult breathing and swelled legs. After being placed in bed his breathing became more and more embarrassed, and he died six hours after admission.

A *post-mortem* examination of the body, eighteen hours after death, shewed effused blood in the muscles of the abdomen, and along the course of the veins at the lower part of the neck: there were also

*It may be well to mention here, that disease has been much more common than usual amongst cattle in the neighbourhood of Carlisle, and, as in the human subject, much less amenable to treatment. Veterinarians have not been able to specify the nature of the epidemic, beyond mentioning the great frequency of *Pleuropneumonia.* As far as I can learn, the "bleeding plan" which ought to obviate pneumonic disease is losing credit, and not a few of the sensible class of farmers, seeing the fallability of the art, eschew physic entirely, and commit their animals to the more tender mercies of nature. It seems pretty certain, however, that the cause of disease amongst the brute creation is not as yet traced to any error in food, whilst the prevailing opinion is in favour of atmospheric influence aiding, if not originating morbid action.

patches of ecchymoses over the convexity of the transverse colon; but these and incipient granular degeneration of the kidneys were the only morbid appearances. The state of the gums and the knee-joints, and a more detailed history from his companion, proved his disease to have been scurvy. The long journey and great exertion were more than his debilitated system could bear up against,—hence the suddenly fatal termination.

My attention was now fully awakened to the subject, and the continual accession of cases from the Caledonian Railway, and out-door applicants in the persons of laborers, shoemakers, weavers, factory-girls, &c., afforded ample scope for investigating the disease in all its relations. I was fortunate also to secure the co-operation of medical friends in the neighbouring towns and rural districts, only two of whom had seen scurvy previously, and some of them naturally had their doubts as to the supervention of a disease which ranked rather amongst "the things that were," in the eyes of landsmen.

So much has been written on the history of scurvy, that I do not feel myself justified in doing more than state that the symptoms presented the true features of Scorbutus, or Sea-Scurvy, such as have been already given in the June number of this journal, p. 878. As worthy of notice I may mention, that hemorrhage was common from the gums. Epistaxes was less frequent. In two cases blood was passed *per anum*, and in one case, but only in trifling quantity, from the bladder. A tendency to menorrhagia was also observed. The stiffness of the joints completely disabled a large number from walking, whilst pains in the joints were at times unusually severe. The embarrassed breathing on the slightest exertion does not seem to have been so particularly noticed on the present epidemic. It was well marked in the severe cases which came under my observation, and to show how rapidly it was induced, a patient in bed attempting to lay hold of an object rather beyond his reach, had a fit of difficult breathing, which lasted an hour and a half. There was nothing abnormal in the respiratory sounds in any of the cases. The urine occasionally showed uric acid. It was difficult to say whether the gums or limbs were first affected.

These remarks are meant to embrace a general survey of what has been observed of scurvy, in a district presenting very varied circumstances in reference to the locality and dwellings, no less than the occupation and living of the individuals affected. What can present a greater contrast in the artizanship of life than the hand-loom weaver of pallid complexion and slender frame, worried with a large family, ill-fed and worse clothed, following his trade in low confined apartments, often in close proximity to noxious animal effluvia, and the railway-labourer, active, athletic, iron-framed, generally without a wedded charge, and the "little responsibilities," well fed, and warmly clothed, and with his avocations inhaling the "incense-breathing morn?" These men may be almost viewed in the light of antipodes to each other in this life of labour; yet they appear to suffer equally from any infringment of that law which points out variety of food as essential to the maintenance of healthy action in the human economy.

To make these remarks as concise as possible, it seems advisable to arrange in different groups the persons affected, treating of the prominent facts in each group, *e. g.* the habits and food previous to the attack, the duration of the disease, and the result of medicinal agents employed in the treatment.

It is by no means an easy matter to arrive at correct data regarding the exact quantity of food used by any class of people, whilst those who depend upon manual labour for their subsistence, will naturally in periods of scarcity be obliged to make the kind and quality subservient to their means.

In Carlisle and its immediate vicinity, the persons chiefly afflicted were weavers, and their wives and daughters, working in the factories, shoemakers, and comparatively few of any other class of artizans. They lived in inferior dwellings, complained of the severity of the winter, want of clothing, and short allowances of food. Bread, oatmeal, treacle in very small quantities, tea and coffee with an occasional herring, formed their entire food. None had tasted potatoes after the harvest of 1846, or for a period of seven or more months, excepting one who remembered Christmas day, from the fact of potatoes forming his principal meal on that festival of the Catholic Church.* The bread was variable in quantity, but never exceeded the demands of the appetite,--in short their food was deficient in quantity, miserably so in variety, and altogether incompatible with the maintenance of vigorous health and strength. Few had made use of milk to any extent, even in the most favourable circumstances for obtaining it, whilst the majority said they had no liking for milk, or that it did not suit them. I was particularly careful in my inquiries on this subject, and from my knowledge of the kind of diet generally adopted by this class of people, the information obtained did not appear remarkable. The lower classes here do not sufficiently appreciate the value of milk, viewing it rather in the light of a drink or vehicle for solid food, and therefore too expensive, except in the rearing of children. On being closely questioned as to the cause of their ailments, not a few adduced a "different living," the want of animal food, but the most striking circumstances in their diet was the want of potatoes, which had been the staple food at dinner, and often at supper. The deficient supply of this esculent had been manifest for two years previous, but never entirely failed till the last season. It therefore became less a matter of surprise that the more intelligent of our patients attributed their unhealthy state to a deficiency of the potato crop. Before offering any decided opinion on this subject, let me remark, that women debilitated with suckling, or of a menorrhagic tendency,—people of

*St. Jerome and the early Fathers might have anathematized the person guilty of such gastronomical remembrance of a holy festivity; in our days, however, the "dignitaries of the Church" appreciate too highly the

"Fair round belly with good capon lined,"

to interfere with the *stomachic* privileges of her majesty's lieges.

both sexes suffering from sores and burns, or who had taken two or three doses of mercury, or laboured under disease which enfeebled the constitution, seemed peculiarly liable to the inroads of scorbutus. Whatever lessened the vital power, developed or hastened on the disease.

This cannot be said of the railway excavators, who in general lived on beef or mutton, salt bacon, suet puddings, bread and butter, oatmeal porridge and treacle, tea and coffee, and occasionally potations of ale or beer. The exact quantities devoured by these muscular fellows could not be ascertained, but that there was no deficiency of the food used is pretty evident from their repeated statements. A personal inspection* of their breakfast and dinner tables enabled me to verify these statements. They assured me that they often consumed a pound of beef at a meal.

The barren mountainous country through which the Caledonian Railway passes for a long distance, precludes the purchase of milk during any part of the year, whilst in the more cultivated districts from Lockerbie southwards, the supply is so scanty that it can hardly be viewed as forming part of the dietary of the excavators.

On questioning these men as to their fondness for, or use of milk, during periods antecedent to their being engaged on this line, I learned that none had been in the habit of using it for the last five years, or so rarely as not to be noticed in any inquiry *quoad* their food. A majority of these excavators were Englishmen belonging to the northern counties. As they constituted a large body of men foreign to the district in which they carried on their labours, the farmers did not consider it politic to increase their stock of cows for a temporary purpose, moreover the rural population were generally intimidated by the outrageous behaviour of these workmen, and did not solicit their favours. Taking these circumstances into consideration, it is reasonable to infer that the quantity of milk consumed by these people was of very small amount. The replies elicited by my repeated questioning confirmed the above view; and the report of Dr. Smith and Mr. Cockburn, of Moffat, is to the same effect. Mr. C. writes, "few of those affected have been in the habit of using milk."

In the most southern part of Dumfriesshire, the Gretna district, scurvy has been very prevalent since January; indeed, I believe that

*I visited the numerous huts from Moffat to the "summit" on Sunday the 2d May, along with Messrs. Thomas and Cockburn. In a great number of the huts we saw the men breakfasting on beefsteaks or mutton chops and bread. The dinner comprised bread, boiled beef, or bacon, pea soup or broth, and suet puddings containing currants. The animal food was taken in large quantities, though the men had not had the benefit of labour or exercise (owing to the day and the heavy rains,) to improve the appetite. I occasionally found the bread stale, and the butter rancid; the "tommy shops" were heavily complained of. The uncooked beef and mutton seemed good, perhaps less fat than usual. The sleeping apartments were much too small, ceilings low, and ventilation imperfect.

The site of the huts was not well chosen, either they were too near the "Avon stream," or on mossy wet soil. In the last mentioned sites, fever made greatest ravages.

here it showed itself sooner than in any part of the wide locality referred to in these remarks. Here the farms are large, and agriculture is conducted with great skill. The land is not much above the level of the high-water mark of the Solway Firth, as it runs up the "Kirtle" and "Sark" rivers. The villages of Gretna, Springfield, and the Rigg of Gretna, are closed to the English borders, and consist of a few straggling houses, inhabited mainly by weavers and field labourers.

In this, as in all rural districts, the farm-servants and cottars have milk twice a day at least, but the other division of the labouring population, the artisans and families, are not favoured with milk beyond the summer and autumnal months; the rest of the year they use treacle-beer to their porridge. In this district, through the kindness of Mr. Carruthers, surgeon, I had the opportunity of observing well-marked cases in both sexes, in cottars who were in the habit of taking milk daily, to the extent of from 10 to 32 ounces; and in artizans, who were obliged to use treacle-beer in lieu of milk for eight months in the year. Mr. Carruthers informed me that potatoes have always formed a staple article of food in this district, comparative little animal food being used, fresh fish and salt bacon occasionally. Oatmeal in various forms is daily consumed. During three winters the potato crop has been failing, but the supply was never entirely cut off till last autumn and winter. In the case of milk there had been no manifest deficiency. Here then, in the Gretna district, a number of people, pretty nearly 100 in all, who, in respect to locality, dwellings, and labour, were the same in 1846–7 that they had been for years previously; but being deprived of that which had long occupied a prominent position in their diet, namely the potatoes, could no longer withstand that breaking down of the constitution which terminated in scurvy. Had the artizan classes been alone affected they would have afforded a strong confirmation of the opinion so ably advocated by Christison; but the fact of many of the agricultural class, who were daily in the habit of using milk, being equally diseased, makes the cause, if an error of diet, (and there is no other apparent cause,) to rest with the potatoes. The following cases may be cited as additional instances to those recorded above. A gentleman of middle age, highly distinguished for his agricultural attainments, who had not used milk for more than twenty years, became afraid, in September last, of eating potatoes, of which esculent he was formerly fond, lest it should induce disease, and suddenly attached himself to cocoa, arrow-root, and other farinacious food,—the quantity in all being deficient for the healthy standard. In April 1847 scorbutic symptoms appeared, and I prescribed for him oranges, beef tea, and a more liberal allowance of food generally. He was restored to health in a fortnight. A clergman in the same vicinity was also afraid of using the potatoes last autumn. His living in other respects was the same as in other years. He became scorbutic about the latter end of April last.

A sailor in the light-ship off Maryport, on the west coast, placed himself under my care in May. He had not tasted fresh vegetables for more than eight months. In other respects his food was the same last year as it had been for four years previously, during which period he had been a strong healthy person.

An agricultural labourer who abandoned potatoes entirely in August, 1846, continuing his small quantities of milk, and the saccharo-farinaceous food so often alluded to, became so severely affected with scurvy, that he could not be raised to be shaved without fainting; his death was daily expected. Can anything be more striking than these cases, exhibiting the cause of the disease?

Dr. John Graham, of Brampton, nine miles east from Carlisle, reports of his cases of scurvy, "the most general fact respecting the diet is the total want of potatoes and fresh vegetables—then comes the total absence of fresh animal food—salt herrings and bacon being the only substitute for it." Again he says, "It appears that the patients, all of whom were paupers, had in former years had potatoes, and though not accustomed to much milk, the quantity not exceeding three or four ounces daily, had generally more than during last season." In a third of Dr. G.'s cases no milk had been taken for two or more years.

Dr. George Mein describes the food of the scorbutics in Cannobie, a rural district near the English borders, as consisting of tea, coffee, porridge, bread and butter, no meat and no vegetables. The frost had spoiled the turnips and cabbages. Milk he reports to be "scarce every winter in Cannobie." Dr. Weir, an old practitioner in the district, does not think the supply of milk differed at all from ordinary years, and attributes the cause of scurvy to the want of the potato and other fresh vegetables.

Dr. Bogie of Annan, Dumfriesshire, has seen between 90 and 100 cases amongst the pauper class, and railway excavators on the Nithsdale Railway. He had previously seen the disease on board of ships. In reference to the present epidemic, he informs me that he "could trace no connexion between milk and the disease." A number, but the proportion he could not specify, made use of milk in the summer months. The food appears to have been very similar to that recorded above, namely, saccharo-farinaceous; the absence of potatoes and fresh vegetables being very prominent. Dr. Bogie describes the pulse as very languid, and "never emptying itself," and that mercury with chalk improved the pulse by relieving the hepatic circulation and producing bilious stools. Contrary to the experience of writers, he found the physiological action of the mercury necessary, in some cases, where nutritious food, and his favourite remedy,—salicin (10 or 12 grains daily,)—had failed in their ordinary beneficial effects in this disease.

The average duration of the disease under salicin and nutritious diet was rather more than a month.

Dr. Walker of Annan had treated a dozen cases. He attributes the disease to the want of potatoes and fresh vegetables.

My highly esteemed friend, Dr. Dickinson, who has had more experience of scurvy than any one in Cumberland, reports that the disease had not shown itself in Workington (a seaport town of 7000 inhabitants on the west coast,) and assigns as a reason, "that vegetable food was more abundant there than in many situations, particularly turnips, of which large quantities were used."

A few days ago, my talented friend, Dr. Browne, Crichton Institution, Dumfries, told me in conversation, that he had scorbutic cases generally every spring, and that during the present season the disease had been much more common, in all probability, from the want of fresh vegetables, as the parties were not without a fair supply of milk. Dr. Grieve, who joined us in conversation, could not trace any connexion between milk and the disease in his abundant experience of scurvy as now prevailing in Dumfries. Both gentlemen agree as to the absence of potatoes being the chief cause, whilst they were inclined to attribute something to atmospheric or other influences which a more extended experience may unravel.

Though the diet of the scorbutics under my care has been fully considered, it is right to state, in reference to Carlisle and its vicinity, that milk was not quite so abundant amongst the poor in the early part of winter, owing not so much to the diminution of supply occasioned by diseased cattle, or lack of fodder, as the fact of provisions generally being almost double the price of ordinary years, thus compelling the adoption of weak tea and coffee. In former years, for instance 1842–3, &c., the "murrain" was common, and milk was then more scarce, and abandoned by many lest it should induce the same disease in the human system,* yet there was no approach to scurvy during that scarcity of milk. It is, indeed, a striking fact, as an intelligent medical friend writes, "that of all the years under the sun, that marked by a dearth of potatoes should be chosen for the advent of scurvy," and, I may add, amongst a class hitherto considered exempt from its attacks. Whilst this clearly points out something else than the want of milk as the *origo mali* of the present epidemic, I am in no wise doubtful, but a copious supply of this bland nourishment would be, in many cases, a useful antiscorbutic, owing to its possessing *per se* those proximate principles proved by Prout, and others, so essential to nutrition.† And I can fully comprehend its great services in turning the tide in favour of health, when a previous diet had been

*It deserves remark, that the poor are always more prejudiced against doubtful articles of diet than those in more easy circumstances. The cry against "murrain milk" was strong during the period. During the present spring it required great and persevering efforts to persuade the starving hundreds to adopt the use of rice, hominy and other substitutes for potatoes.

†There must be *something* in the potatoes; they enable Irishmen to do a great amount of work. Paddy pays us an annual visit in the harvest, and prefers a "potful o' praties ' to the best roast beef which we can set before him. Butter-milk is very well in its way, but we dont churn butter every day of the week. Don't boil the potato to affect the centre much, and Pat will tell you that the "hard mate" and a pickle of salt, "barring the milk," will do "rite well."

Let chemists look to this. The "heart of a potato" may make an English Liebig of some enterprising fellow.

faulty, either in variety, as seen at Perth, or quantity, as too often met with during last season.

*Treatment.*—I have already spoken of the severity of the cases under my care in the Infirmary, and may further add that not one of them was able to chew animal food, or even soft bread, in addition to their being incapacitated from walking from one room to another. Their appetites were good. They were ordered a pint of beef-tea, a pint of porter, 12 oz. of bread, and the same quantity of milk *per diem*, also ʒi or ʒij of citric acid and lemon-juice with 4 oz. of infusion of gentian and water as an acid drink. They soaked the bread in the beef-tea, and relished the bitter infusion and acid drink amazingly. The worst cases had two oranges daily. Nothing in medicine has delighted me so much as witnessing the happy effects of this plan of treatment. Four excavators were placed in a ward by themselves, labouring under scurvy in the most severe form. On the third day after admission, these fellows began to speak in a cheerful tone. Hemorrhage from the gums had ceased, the joints were less painful and less swollen, and no fresh ecchymoses had shown themselves. On the sixth day, the gums enabled them to chew, and other ameliorations had gone on *pari passu* with the gums. On the ninth day they were all sitting up, and enabled to walk along the room; and on the 12th they became useful in cleaning the walks; and on the 14th day, presented themselves to the committee of the institution, and returned thanks for what one of them, the spokesman of the party, chose to call "a miracle effected on them." The weavers did not make such great progress under the same treatment:—from 20 to 26 days being required to effect a cure in them. The excavator, who accompanied the fatal case, remained in the house 40 days. His diet had been no better than the poorest of our scorbutics, and he was not of the most healthy character.

The average duration of the out-patients to whom an abundant supply of the citric acid and gentian mixture was given, and who dieted as they best could of fresh vegetables, was about 25 days. The smallest alteration of diet had a wonderful influence in checking the disease, so that I am not surprised at the accounts given by Lind, of a "few pickled cabbages now and then" affording immunity from the disease. Those who obtained oranges advanced most rapidly. So much was I convinced of the value of oranges, that on being called to an excavator who had lost more than three pints of blood by epistaxis in less than three hours, and was reduced to extreme weakness both in body and mind, I prescribed oranges *ad libitum*. He was badly nursed, and got weak broth, and little farinaceous food, but his recovery was complete within the twenty days. A woman less severely affected than the majority, was cured with a shilling's worth, about 15 oranges! The lightship sailor who came to me on crutches got well on oranges in 14 days. The agricultural labourer, who could not have his head raised without fainting, was cured with oranges and porter, and citric acid, small quantities of each, in about 15 days.

He was "the miracle" of the district in which he lived, as his friends had given him up for lost.

In the list of treatment adopted by others, I see the mineral and vegetable acids, bark, wine, beef-tea, milk, occasional purgatives and diaphoretics, oranges, potatoes, &c. It is often noted by my obliging friends that the scorbutics only had a few potatoes, or fresh vegetables, or milk, and soon rallied; even a good supply of nettle broth or beer cured slight cases; again and again showing that the slightest change of diet or drink was beneficial, and that in proportion to the nutritious quantity of the food along with variety, was the rapidity of the cure.

As vegetables became plentiful, scurvy disappeared from amongst us. I do not hear of fresh cases anywhere in Cumberland at this date (July 9th.)

*Conclusion.*—On weighing all the circumstances related above, I am led to make the following inferences. 1. That as the vegetable world became more or less blighted, man in common with the higher class of animals suffered, from causes not well understood, but apparently of an epidemic nature, which have deteriorated his condition, and made him the more ready victim to scurvy, fever, &c. 2. That scurvy originates from an error of diet as generally believed,—the occupation, dwellings, &c., sometimes viewed as collateral causes having little or no influence. 3. That a deficiency of potatoes constitutes the chief error of diet, and is the main cause of the present epidemic, whilst the absence of variety and deficient quantity of food hastened the development of scurvy. 4. That the use of milk, as might be anticipated from its proximate principles, lessened the liability to scurvy, but did not prevent its occurrence:—its powers in correcting a monotonous diet have been acknowledged in the list of remedial agents.

P. S.—The present epidemic of scurvy contains a warning, which concerns the government, the profession, and the public. Let us hope that it will not be lost sight of.—*Monthly Journal of Med Sci.*

